# Use of Multifrequency Bioimpedance Analysis in Male Patients with Acute Kidney Injury Who Are Undergoing Continuous Veno-Venous Hemodiafiltration

**DOI:** 10.1371/journal.pone.0133199

**Published:** 2015-07-17

**Authors:** Harin Rhee, Keum Sook Jang, Min Ji Shin, Jang Won Lee, Il Young Kim, Sang Heon Song, Dong Won Lee, Soo Bong Lee, Ihm Soo Kwak, Eun Young Seong

**Affiliations:** 1 Department of Internal Medicine, Pusan National University School of Medicine, Busan, Republic of Korea; 2 Biomedical Research Institute, Pusan National University Hospital, Busan, Republic of Korea; 3 Department of Nursing, Pusan National University Hospital, Busan, Republic of Korea; Medical University of Graz, AUSTRIA

## Abstract

**Introduction:**

Fluid overload is a well-known predictor of mortality in patients with acute kidney injury (AKI). Multifrequency bioimpedance analysis (MF-BIA) is a promising tool for quantifying volume status. However, few studies have analyzed the effect of MF-BIA-defined volume status on the mortality of critically ill patients with AKI. This retrospective medical research study aimed to investigate this issue.

**Methods:**

We retrospectively reviewed the medical records of patients with AKI who underwent continuous veno-venous hemodiafiltration (CVVHDF) from Jan. 2013 to Feb. 2014. Female patients were excluded to control for sex-based differences. Volume status was measured using MF-BIA (Inbody S20, Seoul, Korea) at the time of CVVHDF initiation, and volume parameters were adjusted with height squared (H^2^). Binary logistic regression analyses were performed to test independent factors for prediction of in-hospital mortality.

**Results:**

A total of 208 male patients were included in this study. The mean age was 65.19±12.90 years. During the mean ICU stay of 18.29±27.48 days, 40.4% of the patients died. The in-hospital mortality rate increased with increasing total body water (TBW)/H^2^ quartile. In the multivariable analyses, increased TBW/H^2^ (OR 1.312(1.009-1.705), p=0.043) and having lower serum albumin (OR 0.564(0.346-0.919, p=0.022) were independently associated with higher in-hospital mortality. When the intracellular water (ICW)/H^2^ or extracellular water (ECW)/H^2^ was adjusted instead of the TBW/H^2^, only excess ICW/H^2^ was independently associated with increased mortality (OR 1.561(1.012-2.408, p=0.044).

**Conclusions:**

MF-BIA-defined excess TBW/H^2^ and ICW/H^2^ are independently associated with higher in-hospital mortality in male patients with AKI undergoing CVVHDF.

## Introduction

Acute kidney injury (AKI) is common in the intensive care unit (ICU) [1, 2] and often requires renal replacement therapy (RRT) [1, 3]. Despite technical advances in the management of AKI over the last several years [4, 5], ICU mortality is still high at approximately 40 to 50%. Several factors were reported to predict mortality in AKI patients [1, 6], and fluid overload at the time of RRT is a well-known predictor of patient survival [1, 7–9]. In previous studies, fluid overload was quantified as an arithmetical calculation: the sum of the daily fluid intake minus the total output adjusted by the body weight. Although this type of quantification is the easiest and most basic method of assessing volume status, this method is not applicable unless a detailed record of input and output status is available. In addition, this method calculates only the excess total body water (TBW) and cannot differentiate water excess in individual water compartments.

Bioimpedance analysis had long been used in the measurement of the nutritional part of body composition, such as fat mass or fat-free mass in the diverse condition [10, 11]. Recently it has been used as a promising tool for the measurement of volume status [12]. With the electrical properties of body tissues [11, 13, 14], multifrequency-bioimpedance analysis (MF-BIA) differentiates extracellular water (ECW) or intracellular water (ICW) from total body water (TBW) using different frequencies: 0, 1, 5, 50, 100, 200, 500 or 1,000 kHz [13, 14]. ECW is quantified using the data obtained from low frequencies (e.g., 1 or 5 kHz), and TBW is quantified using the data from higher frequencies (e.g., 200, 500 or 1,000 kHz) [15, 16]. ICW can be assessed by subtracting the values measured from the two water compartments [16].

To date, several studies have demonstrated the accuracy and clinical usefulness of MF-BIA in chronic hemodialysis or peritoneal dialysis patients [17–19]. Additionally, in a septic AKI patient, MF-BIA was useful in assessing volume status and net fluid removal by continuous veno-venous hemofiltration successfully reduced TBW, ECW and ICW [20]. However, data on the MF-BIA defined volume status and the clinical outcome of AKI patients who are undergoing continuous veno-venous hemodiafiltration (CVVHDF) are limited [20]. We hypothesized MF-BIA-defined volume overload could be a useful predictor of mortality in patients with AKI. This study aims to investigate this issue and further analyze the effect of fluid accumulation in different compartments on in-hospital mortality.

## Methods

### Subjects

This investigation was a single-center, retrospective study based on consecutively collected data from AKI patients who underwent CVVHDF in the ICU between Jan. 2013 and Feb. 2014. All 327 patients were screened for eligibility. We excluded patients who were 18 years or younger and female patients to control for sex-based differences in the interpretation of the MF-BIA-defined volume status. Patients with a peripheral amputation and patients with a cardiac pacemaker or a defibrillator, for whom we were unable to use the BIA analyzer due to these conditions, were also excluded. Approval to perform anonymous analyses of routinely collected clinical data with a waiver of informed consent was obtained from the Pusan National University IRB Committee [E-2014026]. Due to the retrospective study design, the informed consent was exempt from review according to the IRB, and each patient record was anonymized and de-identified prior to analysis.

### Assessment of body fluid volume using MF-BIA

A single well-trained nurse assessed body fluid volume at the time of CVVHDF initiation, according to the standard AKI protocol of our clinic, using an Inbody S20 system (Biospace, Seoul, Korea). Inbody S20 system measures the electrical responses at multiple frequencies between 1 and 1,000 kHz and estimates ECW and TBW in accordance with reactance and resistance [21]. Measurements were obtained in the supine position, using an eight-hand and foot tactile electrode system. The input variables included the patients’ age, sex, height and actual body weight.

### Data collection and CVVHDF management

Demographic, anthropometric and biochemical data were collected at the time of CVVHDF initiation. The MF-BIA-defined volume status was expressed in liters and normalized based on height squared. We reviewed the indications for CVVHDF initiation, assessed a degree of organ dysfunction using the Sequential Organ Failure Assessment (SOFA) score and disease severity was scored using the Acute Physiology and Chronic Health Evaluation (APACH) II and the Simplified Acute Physiology Score (SAPS) II. We also reviewed the CVVHDF-associated treatment history. The decision to start renal replacement therapy was made when the urine output continued to decrease for more than 12 hours, despite all efforts to control for a correctable cause of AKI or when the medically uncontrolled pulmonary edema or acidemia was defined. CVVHDF was selected as a RRT modality in those situations with hemodynamic instability, acute brain injury or generalized brain edema. The CVVHDF initiation time was assessed as the time from admission to the ICU to the CVVHDF application. Vascular access was achieved via the internal jugular vein or the femoral vein. CVVHDF was performed in all patients using a Prismaflex with AN 69 ST membrane. Heparin was used as an anticoagulant in most cases, and nafamostat mesilate was used in patients with a tendency toward increased bleeding. Hemosol was replaced using pre- and post-dilution methods at a proportion of 2:1. The initial blood flow rate was 100 mL/min, and the blood flow was increased to 150 mL/min based on the patient’s condition. The dose of CVVHDF was prescribed as 40 mL/kg/hr due to the frequent discontinuation of CVVHDF, and the actual delivered dose was calculated using the effluent flow rate with a correction for the percentage of predilution.

### Patient outcome

In-hospital mortality was identified during a medical chart review. We defined in-hospital mortality as death during the hospital stay, including death in the general ward after discharge from the ICU.

### Statistical Analysis

The data were analyzed using SPSS for Windows, version 17.0 (SPSS Inc., Chicago, IL, USA). For continuous variables, the mean ± standard deviation was used to describe normally distributed data, and other data were described using the median. BIA-measured parameters, such as resistance (R), reactance (Xc) and impedance (Z) were normalized with height (H) and BIA-defined ICW, ECW and TBW were normalized based on height squared (H^2^) [22]. Based on the initial TBW/H^2^, we classified the patients into quartiles: 1^st^ quartile, TBW/H^2^<12.5 L/m^2^; 2^nd^ quartile, 12.5 L/m^2^≤TBW/H^2^<14.2 L/m^2^; 3^rd^ quartile, 14.2 L/m^2^≤TBW/H^2^<15.5 L/m^2^ and 4^th^ quartile, TBW/H^2^≥15.5 L/m^2^. The bioimpedance vector analysis (BIVA) is displayed graphically, integrating R/H (in Ohm/m) to Xc/H (in Ohm/m), as previously described [23, 24]. Differences among the four groups were tested using the one-way ANOVA test for continuous variables and chi-square tests for categorical variables. We performed the Pearson correlation test to evaluate the parameters that correlated with TBW/H^2^. To test for trends in in-hospital mortality with increasing levels of TBW/H^2^, we used a linear by linear association. The choice of which variables to include in the equation was based on the results of univariable analyses, where each parameter and in-hospital mortality have a demonstrated association (p<0.1). We also included variables based on empirical evidence where definitive association between in-hospital mortality and an independent variable has been demonstrated in previous studies. Factors that fell under the BMI and SOFA scores were excluded during the adjustment to avoid overlapping. Finally, the following factors were adjusted in the multivariable analyses: age, BMI, the presence of sepsis [25], TBW/H^2^, SOFA score, actual CVVHDF dose [26, 27], CVVHDF initiation time, prothrombin time (PT) and serum albumin levels. Binary logistic regression analyses were performed to find out independent factors in predicting in-hospital mortality. To avoid multicollinearity or near-linear dependence among the regression variables, we tested the variance inflation factor (VIF), and variables that had a VIF value greater than 10 were analyzed in the separate model. P values less than 0.05 were considered to be statistically significant.

## Results

### Patient characteristics

**Fig 1:**
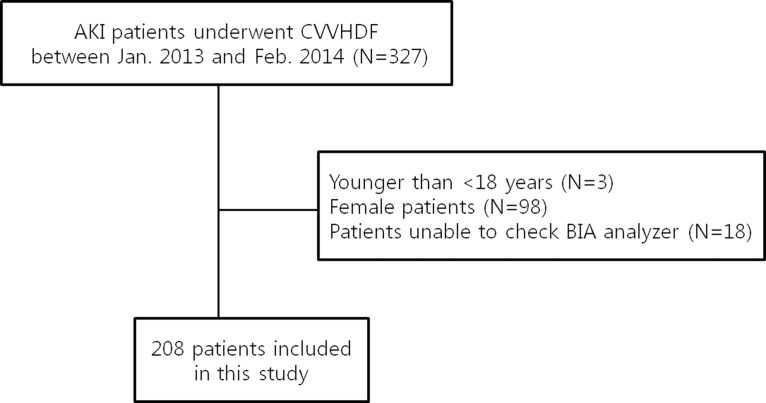
Summary of study flow.

A total of 208 patients were analyzed in this study (Fig 1). The mean age was 65.19±12.90 years, and the mean BMI was 22.62±3.21 kg/m^2^. At the time of ICU admission for the treatment of AKI, the mean SOFA score was 10.61±3.98, and CVVHDF was initiated a mean of 1.38±3.06 days after admission to the ICU. All of the patients were prescribed CVVHDF at a target dose of 40 mL/kg/hr; the mean delivered dose was 31.62±9.72 mL/kg/hr. Septic shock was the underlying disease most probably leading to AKI (Table 1). When we compared the patient characteristics according to the TBW/H^2^ group, weight and BMI were lowest in the 1^st^ quartile. ICW/H^2^ and ECW/H^2^ exhibited an increasing trend in accordance with the TBW/H^2^ quartile. Disease severity, the presence of septic AKI and laboratory values were not different between the groups, and the treatment intensities were similar except for the actual delivered dose of CVVHDF. Detailed information concerning the patient characteristics and treatment history is provided in Table 2.

**Table 1:** Cause of CVVHDF initiation.

Causes	%
**Oliguric AKI**	**68.3**
Septic shock associated with AKI	40.9(85/208)
Oliguric AKI other than sepsis	
Rhabdomyolysis	9.6(20/208)
Tumorlysis syndrome	7.2(15/208)
Ischemic ATN	5.3(11/208)
Drug induced AKI	2.9(6/208)
contrast induced nephropathy	1.4(3/208)
Post renal AKI	0.9(2/208)
**AKI with medically uncontrolled pulmonary edema**	**19.2**
Refractor heart failure	10.6(22/208)
Acute coronary syndrome	7.2(15/208)
Nephrotic syndrome	1.4(3/208)
**AKI with increased intracranial pressure**	**7.2**
Acute brain injury	4.3(9/208)
Generalized brain edema	2.9(6/208)
**AKI with medically uncontrolled metabolic acidosis**	**5.3**
Diabetic ketoacidosis	1.4(3/208)
Alcoholic ketoacidosis	1.4(3/208)
**AKI caused by drug intoxication**	**2.4**
Ethylene glycol	1.4(3/208)
Lithium	0.9(2/208)

Foot note: In most of the cases, the indications of initiating CVVHDF were more than one, and we described most potent indication among them. Oliguria is defined as urine output <0.5ml/kg/hr for ≥12 hours. Abbreviations: AKI, acute kidney injury; CVVHDF, continuous veno-venous hemodiafiltration.

**Table 2:** Patient characteristics, biologic data and treatment according to the quartiles of TBW/H^2^.

	Total (N = 208)	1^st^ quartile (N = 52)	2^nd^ quartile (N = 52)	3^rd^ quartile (N = 52)	4^th^ quartile (N = 52)	P
**Dermographics**						
Age, year	65.19±12.90	66.61±10.98	66.00±13.61	65.28±13.68	62.80±13.38	0.623
Height,cm	168.26±6.35	167.09±5.11	167.75±7.19	168.71±3.93	169.54±6.01	0.390
Weight.kg	64.17±10.54	55.81±8.58	60.75±8.61	67.47±7.39	72.98±8.99	<0.001
BMI.kg/m^2^	22.62±3.21	20.01±3.11	21.59±2.75	23.68±1.84	25.33±2.22	<0.001
**Disease severity and volume status**						
MAP, mmHg	79.50±16.71	81.44±15.31	77.43±14.34	81.95±19.05	77.32±18.01	0.515
Vasopressor, %	62.9	58.3	55.6	61.1	77.1	0.236
SOFA score	10.61±3.98	8.93±2.97	10.51±4.05	11.00±3.84	11.86±4.44	0.025
APACHII score	24.58±6.43	24.38±6.23	23.57±6.03	25.00±6.97	25.43±6.62	0.656
SAPSII	55.13±18.97	53.00±17.67	52.48±17.52	53.03±21.81	61.37±18.06	0.157
Septic AKI, %	40.0	45.7	48.6	31.4	34.3	0.377
Initial ECW/TBW	0.409±0.018	0.412±0.011	0.408±0.013	0.412±0.014	0.406±0.028	0.351
TBW/H^2^,L/m^2^	14.12±2.22	11.33±0.87	13.39±0.48	14.91±0.38	16.94±1.32	<0.001
ECW/H^2^,L/m^2^	5.77±0.97	4.69±0.39	5.39±0.46	6.13±0.27	6.89±0.82	<0.001
ICW/H^2^,L/m^2^	8.36±1.35	6.65±0.54	7.99±0.46	8.78±0.30	10.05±0.81	<0.001
Phase angle,°	3.97±2.41	3.13±2.44	3.99±1.28	4.25±3.08	4.49±2.38	0.094
**Laboratory value**						
WBC,/uL	14.42±9.60	13.31±8.11	14.06±8.43	16.91±13.27	13.54±7.78	0.391
Hb, g/dL	10.32±2.27	10.08±1.65	10.29±2.51	10.50±2.07	10.41±2.72	0.881
TP, g/dL	5.53±1.06	5.89±0.91	5.41±0.97	5.57±1.23	5.23±1.06	0.057
Albumin,g/dL	2.99±0.67	3.22±0.63	2.94±0.72	2.98±0.63	2.85±0.66	0.109
pH, mmHg	7.31±0.13	7.31±0.15	7.32±0.13	7.28±0.11	7.31±0.12	0.613
BUN,mg/dL	54.51±30.73	54.32±28.49	51.48±28.08	57.39±29.71	55.01±36.81	0.889
Creatinine,mg/dL	3.84±2.79	4.08±2.78	3.99±2.51	3.91±3.01	3.40±2.93	0.763
Na,mmol/L	137.52±7.28	136.73±7.34	136.63±6.41	138.37±6.83	138.42±8.47	0.595
K, mmol/L	4.50±1.08	4.62±0.98	4.32±1.09	4.50±1.17	4.59±1.12	0.657
PT, INR	1.56±0.56	1.48±0.39	1.50±0.49	1.63±0.67	1.63±0.64	0.598
**Parameters associated with CVVHDF**						
Initiation time, d	1.38±3.06	1.50±3.70	1.09±1.98	1.22±3.43	1.77±2.92	0.821
Actual dose, mL/kg/hr	31.62±9.72	36.76±10.21	30.33±10.67	29.94±8.31	29.65±8.19	0.008
CVVHDF duration, d	5.21±3.06	4.58±3.52	6.19±5.57	4.22±4.10	5.80±2.03	0.593
Total ICU stay, d	18.29±27.48	18.50±29.33	19.93±29.09	10.25±8.69	24.42±35.63	0.227

Foot note: 1^st^ quartile, TBW/H^2^<12.5 L/m^2^; 2^nd^ quartile, 12.5 L/m^2^≤TBW/H^2^<14.2 L/m^2^; 3^rd^ quartile, 14.2 L/m^2^≤TBW/H^2^<15.5 L/m^2^; 4^th^ quartile, TBW/H^2^≥15.5 L/m^2^. Abbreviations: H, height; BMI, body mass index; MAP, mean arterial pressure; SOFA, sequential organ failure assessment; ECW, extracelluar water; TBW, total body water; ICW, intracellular water; BUN, blood urea nitrogen; TP, total protein; INR, international normalized ratio; CVVHDF, continuous veno-venous hemodiafiltration, ICU, intensive care unit.

### Correlations between the MF-BIA-defined TBW and BIVA

**Fig 2:**
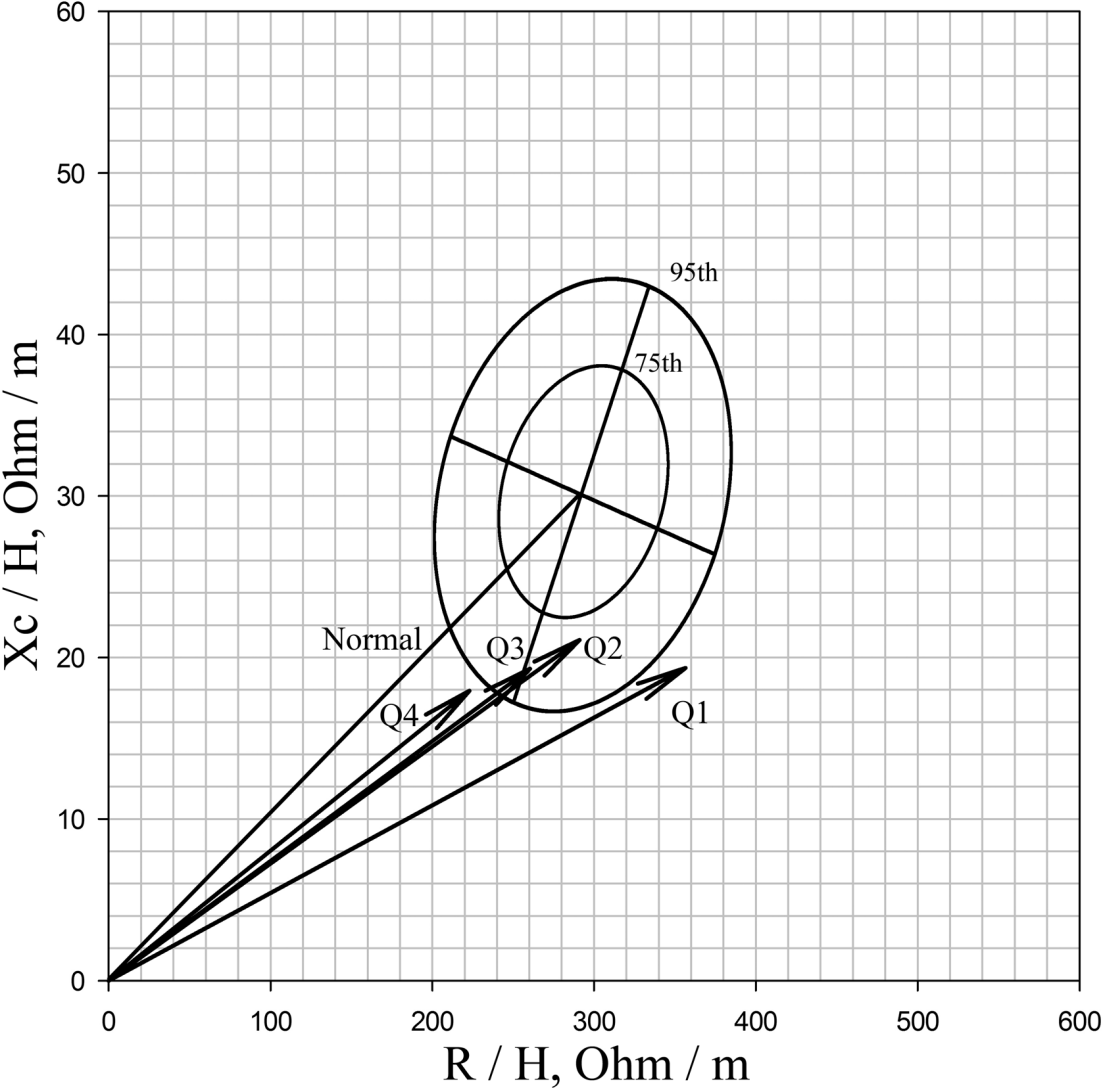
Bioimpdedance vector analysis results of each TBW/H^2^ group displayed graphically comparing resistance/H (R/H) with reactance/H (Xc/H). Male mean impedance/height vector from patients with acute kidney injury undergoing continuous veno-venous hemodiafiltration, plotted on the reference RXc graph [18] with 75th and 95th tolerance ellipses of the male healthy population. Abbreviations: Q1, 1^st^ quartile, Q2, 2^nd^ quartile, Q3, 3^rd^ quartile and Q4, 4^th^ quartile.

TBW/H^2^ was positively correlated with weight and BMI. TBW/H^2^ had an excellent positive correlation with ECW/H^2^ or ICW/H^2^; however, this parameter did not exhibit any relationships with ECW/TBW. Compared to the BIVA data, TBW/H^2^ was negatively correlated with R/H and Z/H at a 50 kHz electrical current. TBW/H^2^ showed a weak positive correlation with the phase angle but did not show any correlations with Xc/H (Table 3). The phase angle showed a strong positive correlation with Xc/H (r = 0.917, p<0.001) and weak positive or negative correlations with ICW/H^2^ (r = 0.227, p = 0.006) or SOFA scores (r = -0.180, p = 0.040). When the BIVA results were displayed graphically, the vector lengths decreased with an increasing quartile of TBW/H^2^(Fig 2).

**Table 3:** Correlations of TBW/H^2^ with anthropometric and MF-BIA defined volume parameters in the critically ill male patients undergoing CVVHDF.

	R	P-value
Age, yr	-0.073	0.385
Height, cm	0.147	0.078
Weight, kg	0.642	<0.001
BMI, kg/m^2^	0.656	<0.001
ECW/TBW	-0.052	0.552
ECW/H^2^, L/m	0.936	<0.001
ICW/H^2^, L/m	0.968	<0.001
50kHz BIVA parameters		
Resistance/H, Ohm/m	-0.788	<0.001
Reactance/H, Ohm/m	-0.083	0.326
Impedance/H vector, Ohm/m	-0.784	<0.001
Phase angle,°	0.183	0.029

Abbreviations: BMI, body mass index; ECW, extracellular water; TBW, total body water; ICW, intracellular water; H^2^, height squared.

### MF-BIA-defined total body water and in-hospital mortality

**Fig 3:**
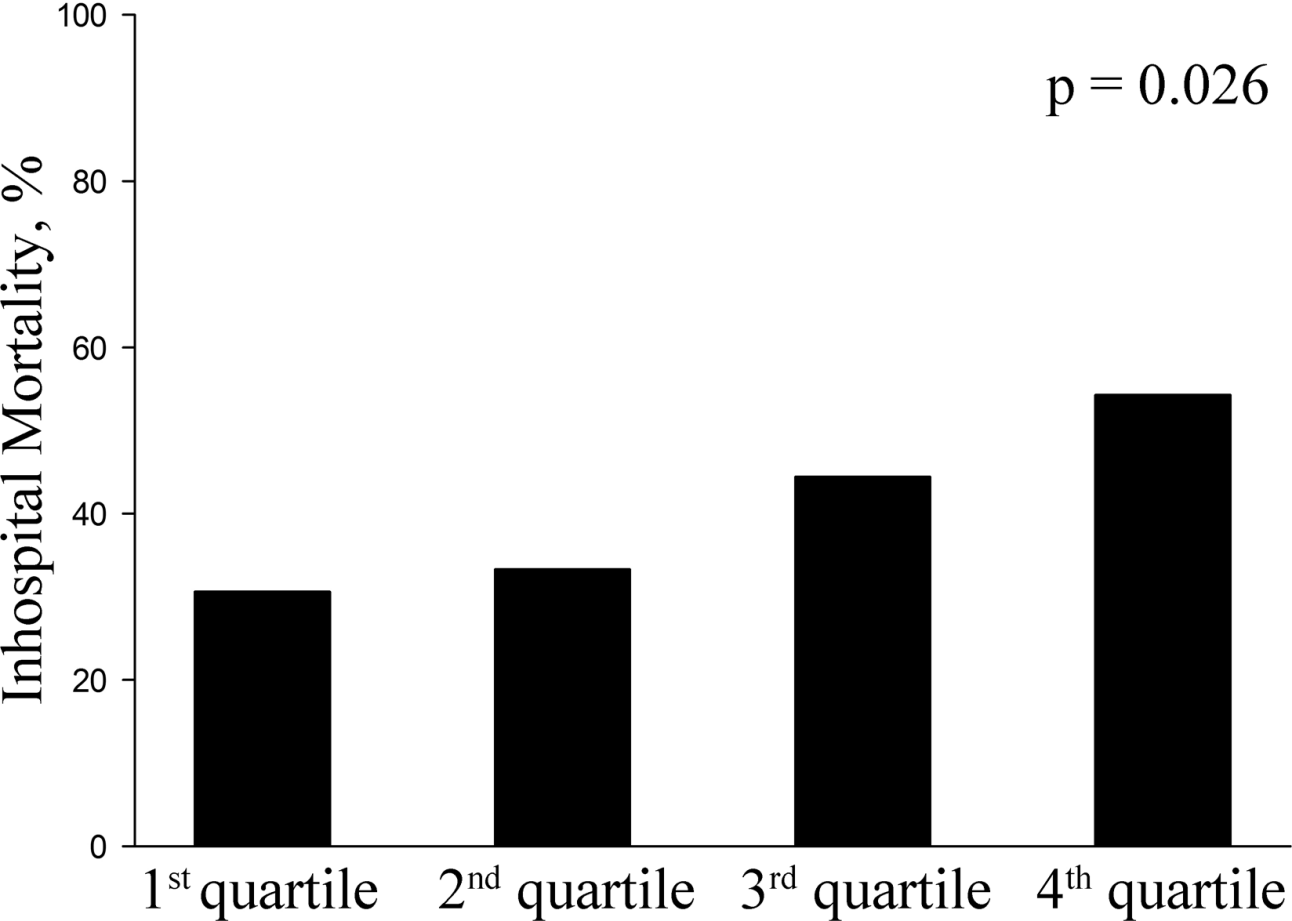
In-hospital mortality showed the increasing pattern in accordance with increasing total body water/height^2^ quartiles in male patients with acute kidney injury undergoing continuous veno-venous hemodiafiltration (CVVHDF). Total body water was measured at the time of CVVHDF initiation, with Inbody S20 (Biospace, Seoul, Korea).

A total of 84 (40.4%) patients died during their hospital stay. As shown in Fig 3, the in-hospital mortality rate increased as the TBW/H^2^ increased. The mortality rates of the four groups were as follows: 1^st^ quartile, 30.6%; 2^nd^ quartile, 33.3%; 3^rd^ quartile, 44.4%; and 4^th^ quartile, 54.3% (p = 0.026) (Fig 3). In the univariable analyses, younger age, heavier weight, larger body fluid (i.e., TBW/H^2^, ECW/H^2^ and ICW/H^2^), higher SOFA scores, higher APACH II score, delayed CVVHDF initiation time, lower serum creatinine and albumin levels, and higher PT values were significantly associated with in-hospital mortality (Table 4). In the multivariable analyses of model I, a larger TBW/H^2^ (OR 1.312(1.0097–1.705), p = 0.043) was independently associated with in-hospital mortality along with lower serum albumin level(OR 0.564(0.316–0.919), p = 0.022) (Table 5). When we further analyzed this adjusting disease severity with the APACH II or the SAPS II instead of the SOFA score, a larger TBW/H^2^ alongside with lower serum albumin levels was still a significant predictor for the in-hospital mortality in this cohort (S1 Table).

**Table 4:** Univariable analysis results predicting in hospital mortality in patients with acute kidney injury requiring CVVHDF therapy.

	In-hospital mortality	
	*HR(95% CI)*	*P-value*
Age, yr	0.976(0.953–0.999)	0.041
Height, cm	1.040(0.992–1.090)	0.101
Weight, kg	1.302(1.004–1.061)	0.025
BMI, kg/m^2^	1.088(0.993–1.193)	0.072
Presence of sepsis	1.495(0.838–2.668)	0.174
ECW/TBW	31.86(0.000–61.03)	0.722
ECW/H^2^, L/m^2^	1.504(1.041–2.173)	0.030
ICW/H^2^, L/m^2^	1.404(1.079–1.827)	0.011
TBW/H^2^, L/m^2^	1.227(1.045–1.442)	0.013
Phase angle,°	0.981(0.853–1.127)	0.784
MAP, mmHg	0.973(0.955–0.991)	0.004
Use of vasopressor,&	3.195(1.723–5.925)	<0.001
SOFA score	1.232(1.126–1.347)	<0.001
APACH II	1.081(1.032–1.133)	0.001
SAPS II	1.011(0.996–1.026)	0.161
CVVHDF initiation time, day	1.046(1.007–1.088)	0.022
CVVHDF dose, mL/kg/hr (actually delivered)	0.988(0.956–1.022)	0.487
WBC,/uL	1.006(0.975–1.038)	0.691
Hb, g/dL	1.009(0.963–1.057)	0.706
Total protein, g/dL	0.462(0.328–0.652)	<0.001
Albumin, g/dL	0.270(0.158–0.462)	<0.001
pH, mmHg	0.114(0.012–1.080)	0.058
BUN, mg/dL	0.998(0.989–1.007)	0.666
Creatinine, mg/dL	0.852(0.757–0.959)	0.008
Na, mmol/L	1.019(0.983–1.056)	0.312
K, mmol/L	0.854(0.663–1.101)	0.225
PT, INR	3.084(1.680–5.662)	<0.001

Abbreviations: BMI, body mass index; ECW, extracelluar water; TBW, total body water; ICW, intracellular water, MAP, mean arterial pressure; SOFA, sequential organ failure assessment; CVVHDF, continuous veno-venous hemodiafiltration; BUN, blood urea nitrogen; Na, sodium; K, potassium; PT, prothrombin time.

**Table 5:** Binary logistic regression analysis results predicting in hospital mortality in patients with AKI requiring CVVHDF therapy.

Model I	OR	P-value
TBW/H^2^, L/m^2^	1.312(1.009–1.705)	0.043
SOFA score	1.130(0.995–1.282)	0.059
CVVHDF initiation time,day	1.036(0.993–1.080)	0.098
Albumin, g/dL	0.564(0.346–0.919)	0.022

In the multivariable analysis age, BMI, the presence of sepsis, TBW/H^2^, SOFA score, actual CVVHDF dose, CVVHDF initiation time, PT and serum albumin levels were adjusted. Abbreviations: CVVHDF, continuous veno-venous hemodiafiltration; TBW, total body water; H^2^, height squared; SOFA, organ failure assessment; BMI, body mass index; PT, prothrombin time.

### MF-BIA-defined intracellular water and in-hospital mortality

Because the Inbody S20 can differentiate ICW or ECW from TBW, we further analyzed harmful hypervolemic effects according to each fluid compartment. The amount of ICW/H^2^ and ECW/H^2^ was positively correlated with TBW/H^2^ (Table 3). To avoid multicollinearity, the effect of compartmental fluid accumulation was analyzed using a separate model. As shown in Table 6, the fluid accumulation in the intracellular compartment was independently associated with in-hospital mortality; however, the fluid accumulation in the extracellular compartment was not an independent predictor of in-hospital mortality when adjustments were made for age, BMI, the presence of sepsis, SOFA score, actual CVVHDF dose, CVVHDF initiation time, PT and serum albumin levels. These results remained same when the disease severity was adjusted with APACH II or SAPS II instead of SOFA score (S1 Table).

**Table 6:** Binary logistic regression analysis results predicting in hospital mortality in patients with AKI requiring CVVHDF therapy.

	Model II		Model III	
ICW/H^2^, L/m^2^	1.561(1.012–2.408)	0.044		
ECW/H^2^, L/m^2^			1.686(0.939–3.027)	0.080
SOFA score	1.102(0.959–1.266)	0.171	1.132(0.997–1.285)	0.056
CRRT initiation time, day	1.038(0.995–1.083)	0.086	1.030(0.990–1.071)	0.144
Albumin, g/dL	0.559(0.343–0.912)	0.020	0.546(0.327–0.912)	0.021

In the multivariable analyses of Model II, age, BMI, the presence of sepsis, ICW/H^2^, sofa score, actual CVVHDF dose, CVVHDF initiation time, PT and serum albumin levels were adjusted. Model III, ICW/H^2^ was replaced to the ECW/H^2^. Abbreviations: CVVHDF, continuous veno-venous hemodiafiltration; BMI, body mass index; ICW, intracellular water; ECW, extracellular water; H^2^, height square; SOFA, organ failure assessment; CVVHDF, continuous veno-venous hemodiafiltration; BMI, body mass index; PT, prothrombin time.

### MF-BIA-defined fluid status in the septic AKI patients

Because septic AKI comprises a large proportion of this cohort (Table 1), we performed subgroup analysis. The most common cause of sepsis was pneumonia, and the second most common cause was colitis (Table 7). We analyzed volume status according to each cause of sepsis; however, it was not significantly different (data not shown) because fluid resuscitation usually preceded CVVHDF and MF-BIA was checked at the time of CVVHDF initiation. When the multivariable analyses were performed, TBW/H^2^ and ICW/H^2^ remained independent predictors of in-hospital mortality along with the delayed CVVHDF initiation time and lower serum albumin level. However, ECW/H^2^ did not predict mortality in the septic male AKI patients (Table 8).

**Table 7:** Underlying conditions of septic AKI.

Pneumonia	50.6(43/85)
Colitis	10.6(9/85)
Panperitonitis	8.2(7/85)
Soft tissue infection	8.2(7/85)
Acute pyelonephritis	7.1(6/85)
Biliary sepsis	7.1(6/85)
Catheter infection	3.5(3/85)
Meningitis	3.5(3/85)
Pancreatitis	1.2(1/85)

Foot note: AKI; acute kidney injury.

**Table 8:** Predictors of ICU mortality in patients with septic AKI undergoing CVVHDF.

	Model I		Model II		Model III	
TBW/H^2^, L/m^2^	1.256(1.006–1.569)	0.044				
ICW/H^2^, L/m^2^			1.476(1.024–2.126)	0.037		
ECW/H^2^, L/m^2^					1.414(0.8446–2.364)	0.186
CVVHDF initiation, day	1.038(1.001–1.077)	0.045	1.041(1.002–1.081)	0.038	1.032(0.996–1.070)	0.084
Albumin, g/dL	0.241 (0.111–0.521)	0.001	0.232(0.107–0.506)	0.001	0.287(0.130–0.635)	0.002

In the multivariable analysis of Model I, age, BMI, TBW/H^2^, SOFA score, actual CVVHDF dose, CVVHDF initiation time, PT and serum albumin levels were adjusted. Model II, TBW/H^2^ was replaced to the ICW/H^2^. Model III, TBW/H^2^ was replaced to the ECW/H^2^. Abbreviations: BMI, bodoy mass index; TBW, total body water; ICW, intracellular water; ECW, extracellular water; H^2^, height squared; CVVHDF, continuous veno-venous hemodiafiltration; BMI, body mass index; PT, prothrombin time.

## Discussion

In this study, we identified MF-BIA-defined TBW/H^2^ excess at the time of CVVHDF initiation as an independent predictor of in-hospital mortality in male AKI patients. With each 1 L/m^2^ increase in the TBW/H^2^, in-hospital mortality increased by 31.2% when adjustments were made for age, BMI, the presence of sepsis, SOFA score, actual CVVHDF dose, CVVHDF initiation time, PT and serum albumin levels in a binary logistic regression analysis. When the patients were divided into quartiles according to the TBW/H^2^ level, the in-hospital mortality rate showed an increasing trend in accordance with TBW/H^2^ quartiles. When the TBW/H^2^ was separated into ICW/H^2^ and ECW/H^2^, excess fluid in each compartment was harmful in the univariable analyses. However, in the multivariable analyses, only excess ICW/H^2^ was an independent predictor of in-hospital mortality and this distinct performance of ICW/H^2^ was maintained in the subgroup analysis of septic male AKI patients.

In chronic hemodialysis (HD) patients, excess fluid accumulates primarily in the extracellular compartment [28, 29]. In Wizemann et al.’s study, the amount of excess ECW was an independent predictor of mortality in chronic HD patients [30], and in Moissl et al.’s study, BIA guided ECW removal improved overall fluid status and blood pressure in HD patients [31]. However, in patients with AKI, excess fluid can accumulate in both the intra- and extracellular compartments due to increased capillary and cell membrane permeability caused by increased inflammatory reactions [32]. Microvascular permeability is particularly increased during sepsis, thus more fluid can be accumulated in the intracellular compartment. In a study of Dabrowski et al., which analyzed body fluid status in septic AKI patients, body fluid accumulated both in the extra and intra-cellular compartment and successful fluid removal in both compartments was associated with favorable clinical outcomes [20]. In the present study, ICW/H^2^ was an important predictor of in-hospital mortality and its performance exceeded the SOFA score, especially in the septic AKI patients. It was still effective when another ICU scoring system, such as the APACH II or the SAPS II, was adjusted instead of the SOFA. Thus, the excess intracellular fluid might be harmful by itself in the septic male AKI patients undergoing CVVHDF.

The integrity of the cell membrane can be assessed based on the phase angle when using MF-BIA [24, 33]. The phase angle is the arc tangent of Xc/R and represents the phase difference between voltage and current [24]. When a current passes through cells, a portion of the electrical current is stored and subsequently released in a different phase [33]. In previous studies, phase angle was known to represent the cellular health, and a lower phase angle was a predictive marker of mortality [34, 35] and a phase angle <5.38° was a significant predictor of mortality in patients with HIV [36]. In the present study, the mean phase angle was 3.93±2.41°, which was relatively lower than the cutoff value of the previous literature. However, in the present study, the phase angle did not show any statistical significance in the prediction of in-hospital mortality. It is tempting to speculate that the phase angle also be affected by the increased ICW/ECW ratio. When the bioimpedance analyzer measures volume status, the electrical current only passes the ionized water compartments within the body and thus the volume of TBW can be estimated from resistance. Reactance reflects the ability of cell membranes to act as imperfect capacitors. Phase angle is the relation between these two vector components of impedance. Therefore, phase angle is affected by the distribution of water between the intra- and extracellular spaces and a high phase angle corresponds to an increased ICW/ECW ratio [37, 38]. The previous studies analyzed the relationship between the phase angle and mortality in a steady state in which ICW/ECW ratio was constant. However, in the present study, the ICW/ECW ratio was different for each of the TBW/H^2^ quartiles and it is possible that the difference of the ICW/ECW ratio observed affects the phase angle in a way that reflects cellular health or mortality reported here.

The electrical data from MF-BIA can be expressed in many ways: raw electrical data can be directly analyzed by vector plot (BIVA) or the raw data could be interpolated into liters of body fluid, adjusting for height and weight (i.e., TBW, in liters) [22]. Although the latter form of data is more intuitive and easy to use, these data can be highly influenced by height or weight, thus they need normalization. In the previous literatures, BIVA data were usually normalized with height [24, 39]. In a similar way, we adjusted MF-BIA defined volume parameters with height but these parameters still had strong correlations with height. To remove this association, we normalized the parameters to height squared and thus could remove their associations with height.

BIVA expresses volume status with raw impedance data that are independent of height or weight. It is an excellent indicator of TBW and the most-verified method in expressing volume status [33, 40]. In the present study, before we used the Inbody—reported TBW, we compared the obtained values with the vector analysis data. In a vector plot, shorter length is associated with increased fluid volume [24]. In the present study, vector length decreased with the increasing quartile of TBW/H^2^. In addition, as shown in previous studies [40–42], the resistance and impedance values adjusted with height exhibited a significant negative correlation with the TBW/H^2^. However, we could not compare this value with the Inbody—reported ICW or ECW because vector analysis data at 50 kHz reflect only the amount of TBW.

The ECW/TBW ratio is an easy and intuitive method for expressing volume status and a well-validated predictor of survival [22]. However, this parameter was not related to the TBW/H^2^ and was not a predictor of in-hospital mortality in the present study. In a steady state, excess fluid usually accumulates in the extracellular compartment. Therefore, increased ratio of ECW/TBW is a good marker for the expression of fluid overload. However, in a critically ill condition such as AKI fluid can be accumulated in the intracellular compartment. Therefore, ECW/TBW can be decreased in the fluid excess condition, if the degree of ICW excess surpasses ECW excess. Thus, this parameter might be better interpreted as a parameter of overhydration only in the steady state.

In the present study, the actual delivered CVVHDF dose varied, although the prescribed dose was consistent at 40 mL/kg/hr according to the CVVHDF protocol of our clinic. In the present study, the doses decreased in accordance with the increasing TBW/H^2^ quartile, which might be due to the more frequent interruption of CVVHDF during Hemosol bag changes in heavier patients. Because the actual delivered dose is an important element of the CVVHDF treatment that affects patient survival [43, 44], a lower dose in the higher TBW/H^2^ group might contribute to lower patient survival. In the present study the actually delivered dose of CVVHDF was low in some patients; the lowest mean dose was 29.65±8.19 mL/kg/hr in 4^th^ quartile, and its efficiency was rather poor. Similar effect of low dose CVVHDF was presented in ATN and RENAL trials [26, 45].

Our data have several limitations. First, we did not consider intra-abdominal pressure, which could falsely increase lower limb venous pressure, thereby overestimating overall fluid volume [46]. Second, we did not calculate the volume excess using the arithmetical method and compare that value with the Inbody-reported data. Third, we did not analyze the MF-BIA-defined volume effect in female patients. Finally, we did not assess the removal of retained ICW or inter compartmental fluid shift during CVVHDF; the clinical outcomes of these approaches should be analyzed in a prospective cohort. However, our study is valuable because it is the first to report on the association between MF-BIA-defined volume status and in-hospital mortality in the context of AKI. In addition, the findings underscore the importance of excess ICW/H^2^ in male AKI patients who are undergoing CVVHDF.

In conclusion, MF-BIA-defined excess TBW/H^2^ measured at the time of RRT initiation is an important determinant of in-hospital mortality in male patients with AKI who are undergoing CVVHDF. Further work based on this result is needed to confirm the importance of ICW/H^2^ excess and the survival benefit of removing this excess during successful CVVHDF treatment.

## Supporting Information

S1 Table Factors associated with in-hospital mortality in patients with AKI requiring CVVHDF according to the different ICU scoring system. (DOCX)

S2 Table STROBE statement checklist of items included in this study. (DOCX)
