# Supplementary material for: Use of Multifrequency Bioimpedance Analysis in Male Patients with Acute Kidney Injury Who Are Undergoing Continuous Veno-Venous Hemodiafiltration
Source: PLoS One. 2015 Jul 17;10(7):e0133199. doi: 10.1371/journal.pone.0133199 (PMC4505923; doi:10.1371/journal.pone.0133199)
Supplement: S1 Table — (DOCX) [file pone.0133199.s001.docx]

S1Table. Factors associated with in hospital mortality in patients with AKI requiring CVVHDF according to the different ICU scoring system

1. APACH II

|  | **Model I** |  | | | **Model II** | |  | | **Model III** | |
| --- | --- | --- | --- | --- | --- | --- | --- | --- | --- | --- |
| TBW/H^2^, L/m^2^ | 1.276(1.019-1.597) | | 0.034 |  | |  | |  | |  |
| ICW/H^2^, L/m^2^ |  | |  | 1.482(1.026-2.139) | | 0.036 | |  | |  |
| ECW/H^2^, L/m^2^ |  | |  |  | |  | | 1.672(0.989-2.824) | | 0.055 |
| APACH II | 1.068(0.994-1.147) | | 0.072 | 1.069(0.995-1.148) | | 0.069 | | 1.068(0.994-1.147) | | 0.073 |
| CVVHDF initiation time,d | 1.039(0.997-1.084) | | 0.068 | 1.041(0.998-1.086) | | 0.063 | | 1.031(0.992-1.071) | | 0.122 |
| Albumin, g/dL | 0.543(0.343-0.859) | | 0.009 | 0.539(0.340-0.853) | | 0.008 | | 0.539(0.334-0.871) | | 0.012 |
| 1. SAPS II |  |  | | |  | |  | |  | |
|  | **Model I** |  | | | **Model II** | |  | | **Model III** | |
| TBW/H^2^, L/m^2^ | 1.283(1.017-1.618) | | 0.035 |  | |  | |  | |  |
| ICW/H^2^, L/m^2^ |  | |  | 1.495(1.023-2.183) | | 0.038 | |  | |  |
| ECW/H^2^, L/m^2^ |  | |  |  | |  | | 1.654(0.964-2.838) | | 0.068 |
| SAPS II | 1.015(0.991-1.040) | | 0.211 | 1.016(0.992-1.040) | | 0.198 | | 1.019(0.994-1.044) | | 0.138 |
| CVVHDF initiation time,d | 1.044(1.001-1.089) | | 0.043 | 1.046(1.002-1.091) | | 0.040 | | 1.034(0.994-1.075) | | 0.093 |
| Albumin, g/dL | 0.573(0.361-0.908) | | 0.018 | 0.568(0.358-0.902) | | 0.017 | | 0.558(0.0.341-0.914 | | 0.020 |

A) In the multivariable analysis of Model I, age, BMI, the presence of sepsis, TBW/H, APACH II score, actual CVVHDF dose, CVVHDF initiation time, PT and serum albumin levels were adjusted. Model II, TBW/H^2^ was replaced to the ICW/H^2^. Model III, TBW/H^2^ was replaced to the ECW/H^2^. B) Disease severity was scored by SAPS II instead of APACH II. Abbreviations: BMI, body mass index; TBW, total body water; ICW, intracellular water; ECW, extracellular water; H^2^, height squred; CVVHDF, continuous veno-venous hemodiafiltration; PT, prothrombin time
